# Symptom Severity Following Rifaximin and the Probiotic VSL#3 in Patients with Chronic Pelvic Pain Syndrome (Due to Inflammatory Prostatitis) Plus Irritable Bowel Syndrome

**DOI:** 10.3390/nu9111208

**Published:** 2017-11-03

**Authors:** Enzo Vicari, Michele Salemi, Giuseppe Sidoti, Mariano Malaguarnera, Roberto Castiglione

**Affiliations:** 1 Section of Endocrinology, Andrology and Internal Medicine, Department of Clinical and Experimental Medicine, University of Catania, 95123 Catania, Italy; enzodante@email.it (E.V.); robertocastiglionen@tiscali.it (R.C.); 2 IRCCS Oasi Institute for Research on Mental Retardation and Brain Aging, Via Conte Ruggiero 73, 94018 Troina, Italy; Micezia@tiscali.it; 3 UOSD Medicina Interna Ambulatorio Andrologia & Endocrinologia ARNAS—Garibaldi, 95123 Catania, Italy; gbsidoti@alice.it; 4 Research Center “The Great Senescence”, University of Catania, 95100 Catania, Italy

**Keywords:** chronic pelvic pain syndrome, irritable bowel syndrome, irritable bowel syndrome-severity scoring system, rifaximin, probiotic VSL#3

## Abstract

This study investigated the effects of long-term treatment with rifaximin and the probiotic VSL#3 on uro-genital and gastrointestinal symptoms in patients with chronic prostatitis/chronic pelvic pain syndrome (CP/CPPS) plus diarrhoea-predominant irritable bowel syndrome (D-IBS) compared with patients with D-IBS alone. Eighty-five patients with CP/CPPS (45 with subtype IIIa and 40 with IIIb) plus D-IBS according to the Rome III criteria and an aged-matched control-group of patients with D-IBS alone (*n* = 75) received rifaximin and VSL#3. The primary endpoints were the response rates of IBS and CP/CPPS symptoms, assessed respectively through Irritable Bowel Syndrome Severity Scoring System (IBS-SSS) and The National Institute of Health Chronic Prostatitis Symptom Index (NIH-CPSI), and performed at the start of therapy (V0) and three months after (V3). In IIIa prostatitis patients, the total NIH-CPSI scores significantly (*p* < 0.05) decreased from a baseline mean value of 21.2 to 14.5 at V3 , as did all subscales, and in the IIIb the total NIH-CPSI score also significantly decreased (from 17.4 to 15.1). Patients with IBS alone showed no significant differences in NIH-CPSI score. At V3, significantly greater improvement in the IBS-SSS and responder rate were found in IIIa patients. Our results were explained through a better individual response at V3 in IIIa prostatitis of urinary and gastrointestinal symptoms, while mean leukocyte counts on expressed prostate secretion (EPS) after prostate massage significantly lowered only in IIIa cases.

## 1. Introduction

Over the past decade, the emerging insights of studies on the concomitant presence of some urologic chronic pelvic pain syndromes (UCPPS) (chronic pelvic pain, interstitial cystitis, painful bladder syndrome, prostatitis syndromes (PS), and vulvodynia) and non-urological associated syndromes (fibromyalgia, chronic fatigue syndrome, and irritable bowel syndrome (IBS)) have attracted significant interest by the Multidisciplinary Approach to the Study of Chronic Pelvic Pain research network established by National Institute of Diabetes and Digestive and Kidney Diseases (NIDDK), with the aim of better understanding and evaluating visceral pain and lower urinary tract symptoms associated with UCPPS, and more systemic contributions to the pathophysiology of these disabling syndromes [1]. 

In particular, PS and IBS are functional, somatoform disorders with a high worldwide prevalence estimated at 11–16% [2,3] and 10–20% [4,5], respectively. Recently, we observed the simultaneous presence of PS and IBS in 30.2% and 31.8% of patients screened by andrologists and gastroenterologists, respectively [6,7], which is in agreement with other reports [8,9]. We found that patients with PS plus IBS also had a significantly higher frequency of chronic bacterial prostatitis (CBP) and lower frequency of non-inflammatory prostatitis (IIIb category) compared with patients with PS alone. The frequency of inflammatory prostatitis (IIIa category) had similar results [6].

PS and IBS are both characterised by a multifactorial pathogenesis, and these conditions are defined on the basis of clinical presentation rather than clear diagnostic markers or findings.

In fact, in terms of pathogenetic aspects, IBS includes disorders of the intestinal barrier, motility, secretion, visceral sensitivity, and interactions between psychological and psychosocial factors [10,11], while chronic prostatitis/chronic pelvic pain syndrome (CP/CPPS) is defined as “urologic pain or discomfort in the pelvic region, associated with urinary symptoms and/or sexual dysfunction, lasting for at least three of the previous six months” in the absence of any identifiable pathology such as cancer, culturable infection, or anatomic abnormalities, often accompanied by “associated negative cognitive, behavioural, sexual, or emotional consequences” [12].

On the other hand, in terms of diagnostic aspects, the diagnosis of IBS requires the administration of the Rome III questionnaire, while PS is identified through the National Institute of Health’s Chronic Prostatitis Symptom Index (NIH-CPSI).

The main diagnostics of the Rome III criteria as established by international professional organisations are based on exclusion criteria and the occurrence and rate of symptoms. In particular, IBS is defined by the Rome III criteria as an abdominal pain or discomfort, often associated with defecation, and with at least two of the following features: altered frequency or consistency, and/or passage of stools, and/or associated feelings of abdominal distension or bloating (10). The symptoms have to be present for at least three months and evidence for an organic underlying cause must be excluded to establish the diagnosis [10,13].

The severity of IBS symptoms are usually measured by using one of the following qualitative instruments: the IBS Severity Scoring System (IBS-SSS), the Bristol Stool Form Scale (BSFS), and the Gastrointestinal Symptom Rating Scale modified for use in patients with IBS (GSRS-IBS) [14,15,16].

On the other hand, the NIH-CPSI, considered the first validated tool for assessing symptom severity in PS, has been proposed for quantifying signs and symptoms and their impact on a patient’s quality of life [17,18].

Despite intensive study over the past decade, clinical trials have failed to identify effective therapies for IBS or CP/CPPS. The efficacy of some probiotics and non-systemic antibiotics (e.g., rifaximin), mainly in infectious IBS, is supported by various evidences [19].

The theoretical basis for simultaneous treatment of the genitourinary and gastrointestinal tract has become more compelling given the evidence of overlapping innervations of the colon and bladder, and the influence of inflammation in one organ on the other [20]. This is why we aimed in the present study to ascertain whether uro-genital and gastrointestinal symptoms of men with CP/CPPS plus IBS may benefit from this three-month therapeutic combination, along with encouragement by recent results of long-term treatment with rifaximin and the probiotic VSL#3, which was effective in lowering the progression of prostatitis into more complicated forms of male accessory gland infections in infertile patients with bacteriologically cured CBP plus IBS [21].

The primary endpoints were the response rate of D-IBS and CP/CPPS symptoms, respectively assessed by using the IBS-SSS (taking a cut-off IBS-SSS reduction level of 50 points as an improvement) [15] and the total scores of the NIH-CPSI, considering as a minimum a six-point reduction in the total NIH-CPSI score [18].

## 2. Materials and Methods

This observational study was conducted at the Andrology and Endocrinology Unit Clinic, Policlinic University of Catania (Catania, Italy), between January 2011 and January 2013. Eighty-five selected male outpatients (median age: 30 years, range: 23–44 years) with a confirmed diagnosis of CP/CPSS plus D-IBS (Rome III criteria) were enrolled in this study, and 75 patients (median age 35 years, range 32–45 years) screened in the same period, affected by IBS alone, served as the control group. The diagnosis of CP/CPPS and D-IBS was made 20–60 months before the patients were included in this study. The protocol was approved by the internal Institutional Review Board of the University of Catania and an informed written consent was obtained from each man. All patients and controls underwent collection of their clinical history, administration of Rome III and NIH-CPSI questionnaires for IBS and prostatitis, respectively, and a physical examination.

Inclusion criteria of patients and the control group were, respectively, diagnosis of CP/CPSS plus D-IBS and IBS alone. Exclusion criteria were as follows: (1) history of chronic bacterial prostatitis (NIH type II) with a positive bacteriological finding at spermculture or at the Meares-Stamey four-glass test [22]; (2) subjects suffering from chronic or acute illness that could interfere with the study, who were taking medications that could interfere in the study (including anti-inflammatory drugs, proton pump inhibitors (PPIs), antidepressants, anti-diarrhoeal, prokinetics, and antispasmodic agents), and who consumed antibiotics or probiotics in the four weeks prior to entering the study; (3) obesity (defined as a body mass index (BMI) greater than or equal to 30 kg/m^2^); (4) subjects affected by major concomitant diseases, with known anatomical abnormalities of the urinary tract or with evidence of other urological diseases, and with residual urine volume >50 mL resulting from bladder outlet obstruction; (5) patients with a history of gastrointestinal bleeding or duodenal or gastric ulcers [23]; and (6) patients that use VSL3 or other probiotic formulations, herbal medicines, or prostatitis treatments [24,25,26,27,28].

### 2.1. Diagnostic Rome III Criteria for IBS

This diagnosis was specifically based on the presence of abdominal pain or discomfort for at least three months in the previous six months, with two or more of the following symptoms: pain improved after defecation, symptoms associated with a change in frequency of stool, and symptoms associated with a change in stool appearance. A simple 10-point objective questionnaire based on the Rome III IBS module was used [18,23]. The presence of a diarrhoea-predominant IBS was found if patients had loose, mushy, or watery stools in the last three months, with no hard or lumpy stools ((question 9 = 0) and (question 10 > 0)) [18,23].

### 2.2. Diagnostic Symptoms Suggestive of CP/CPPS

The symptoms of CP/CPPS were evaluated by the NIH-CPSI questionnaire included for at least three months during the six months before the study, according to the European Association of Urology (EAU) guidelines [29]: pain or discomfort in the pubic or bladder area, perineum, testis, or at the tip of the penis not related to urination; ejaculatory pain; pain or burning during urination, incomplete emptying, and urinary frequency. Their NIH-CPSI pain sub-score was >8 (moderate to severe) [30].

A patient was assigned to the NIH category of the IIIa group if negative bacteriological findings were revealed by the Meares-Stamey four-glass test [31] and the white blood cell (WBC) count in the expressed prostrate secretion (EPS) was equal to or greater than 10 per high power field (HPF) or the WBC count in the VB3 was equal to or greater than 5 per HPF [32]. Conversely, a patient was assigned to the IIIb group if the WBC count in the EPS was less than 10 per HPF or the WBC count in the VB3 was less than 5 per HPF [32].

### 2.3. All Patients Completed the Treatment

#### Treatment Plan

At time-point V0 (visit zero), all patients and control groups were prescribed treatment with rifaximin, a non-absorbable antibiotic (200 mg, 2 tablets bid) (Normix^®^, Alpha Wassermann, Alanno, PE, Italy) for seven days per month for three months followed by a probiotic combination VSL#3 (450 × 10^9^ CFU/day, one small envelope) (VSL#3^®^, Ferring SpA, Milan, Italy).

The probiotic VSL#3 is a mixture of eight different species of Gram-positive bacteria, namely *Streptococcus salivarius* subsp. *thermophilus*, *Lactobacillus casei*, *Lactobacillus plantarum*, *Lactobacillus acidophilus*, *Lactobacillus delbrueckii* subsp. *bulgaricus*, *Bifidobacteria longum*, *Bifidobacteria infantis*, and *Bifidobacteria breve*. The probiotic combination VSL#3 was chosen because it has many interesting properties (reduction in inflammation, antioxidant capacity, and potential benefit for the treatment of IBS; effective in lowering the progression of prostatitis into more complicated forms of male accessory gland infections in infertile patients with bacteriologically cured CBP plus IBS), both in vitro and in vivo that may account for its clinical efficacy [13,21].

### 2.4. Assessment of Symptoms

A follow-up visit, including assessment of symptoms of IBS and CP/CPPS through specific validated questionnaires and urological visits, was performed three months (V3) after the start of therapy (V0).

D-IBS: To monitor D-IBS symptoms and changes, all three groups were asked to register their symptoms weekly using the IBS-SSS questionnaire [16], which includes five items on a 0–100 mm visual analogue scale with total scores ranging from 0 to 500 mm: severity of abdominal pain (Question 1), frequency of abdominal pain (Question 2), severity of abdominal distension (Question 3), dissatisfaction with bowel habits (Question 4), and interference with quality of life (Question 5). This score classifies subjects as having no symptoms (<75), mild (75–174), moderate (175–300), or severe IBS (>300). The primary endpoint was a cut-off IBS-SSS reduction level of 50 points was considered to be an improvement [15]. Secondary endpoints were expressed as individual symptoms of IBS.

CP/CPPS: CP/CPPS symptoms were assessed at V0 and V3 through the NIH-CPSI score. The primary endpoint was a minimum six-point reduction in the total NIH-CPSI score, because it was considered as a clinically appreciable improvement of CP/CPPS symptoms [7]. Secondary endpoints were expressed as individual symptoms of CP/CPPS (NIH-CPSI subscale values); WBC on EPS after prostate massage.

### 2.5. Statistical Analysis

The software SPSS 9.0 for Windows (Chicago, IL, USA) was used for statistical evaluation. Quantitative data were expressed as median and range, and qualitative data were expressed as percentages throughout the study. Intragroup differences in NIH-CPSI or IBS-SSS questionnaire scores before/after therapy were analysed using Wilcoxon’s signed rank test. Mann-Whitney U tests were used for analyses that compared different groups. A statistically significant difference was accepted when the *p* value was lower than 0.05.

## 3. Results

The demographic and baseline characteristics of the participants are shown in Table 1. Age and time since diagnosis were similar in patients with CP/CPPS (prostatitis IIIa and IIIb subtypes) plus D-IBS or D-IBS alone (Table 1). All patients in our study had a normal BMI, which was similar in all three groups. Furthermore, the mean leukocyte counts on EPS after prostate massage were significant (*p* < 0.05) in NIH category IIIa patients plus D-IBS > IIIb patients plus D-IBS > D-IBS alone (Table 1). All patients and controls completed the treatment as planned.

However, the total NIH-CPSI scores significantly (*p* < 0.05) decreased in IIIa patients from a baseline (V0) mean value of 21.2 to 14.5 at V3, as did all subscales (pain, urinary, quality of life), and the total NIH-CPSI score significantly decreased in IIIb patients (from 17.4 to 15.1). In contrast, patients with IBS alone did not show any significant differences in the NIH-CPSI score (total and subscales) (Table 2).

Furthermore, 49.4% of patients (42 out of 85) showed clinical improvement (in terms of a six-point or more reduction in total NIH-CPSI score), with a significant difference between the response rate of NIH category IIIa and IIIb, since a six-point or more reduction in total NIH-CPSI score was found respectively in 71% or 25% of these categories.

Regarding gastrointestinal symptoms, at V0 patients affected by the IIIa inflammatory sub-category of CP/CPPS generally exhibited not statistically significant higher IBS-SSS (mean 298.4, range 180–410) than that registered in IIIb patients (270.0, range 163–388) or the control group (262.5, range 156–397) (Table 2). At V3, the IBS-SSS was significantly reduced in patients with IIIa prostatitis (mean 192.5, range 117–246) compared to that observed in IIIb prostatitis (mean 198.5, range 135–265) or controls (mean 204.0, range 129–266). In patients with IIIa prostatitis, the significant improvement from baseline for IBS-SSS was associated with a responder rate (in terms of decline >50 point) of 77.7% (35 out of 45 patients), significantly higher than the rate values found in IIIb patients (32.5%; 13 out of 40 patients).

Furthermore, regarding the secondary endpoints of the study, we also registered a better individual response in IIIa prostatitis (compared with IIIb prostatitis plus D-IBS or D-IBS alone) of urinary and gastrointestinal symptoms, and mean leukocyte counts on EPS after prostate massage were significantly lowered (from 12 to 7) in IIIa cases only (*p* < 0.05).

### Compliance

All patients completed the treatment. No serious adverse events were reported in the study groups.

## 4. Discussion

Comorbidities are common in IBS and can affect both the gastrointestinal tract, including functional chest pain, heartburn, dyspepsia, and/or abdominal pain [33], and other systems with pain-related disorders, including migraine headache, fibromyalgia, and chronic pelvic pain [34].

In our study, we observed a significant decrease of the total NIH-CPSI and in pain, urinary disorder, and quality of life subscales in IIIa patients and in total NIH-CPS score in IIIb patients; in contrast, no significant differences were observed in patients with IBS alone.

Recently, we showed that PS and IBS co-exist in an elevated percentage (31.2%) of patients who seek medical advice for PS or IBS in andrological or gastroenterological settings, respectively [21]. These patients also had severer urinary (total score and pain subscale) and gastrointestinal symptoms, with significantly higher scores in the NIH-CPSI and Rome III questionnaires compared with patients with PS or IBS alone [21]. These results are in agreement with other reports [8,9].

Despite intensive study over the past decade, clinical trials have failed to identify effective therapies.

In patients with IBS, although probiotics had beneficial effects on global IBS, abdominal pain, bloating, and flatulence scores [35], when considered as a whole, meta-analyses are difficult due to the heterogeneity of the studies of probiotics in IBS and the use of different bacterial strains and different mixtures of these strains, as well as different dosages [36]. Rifaximin was approved in 2015 for the treatment of IBS with diarrhoea, and after two weeks of treatment can result in symptom improvement that persists ≥12 weeks post-treatment [19]; it is by far the best-studied antibiotic in IBS, and it has several appealing properties (gut-specificity; predominant effect intraluminal, limited systemic availability; it does not significantly alter the microbiota of the GI tract; it does not tend to cause diarrhoea or other superinfections such as *C. difficile*). Rifaximin proved more effective than a placebo for global symptoms and bloating in IBS patients [37].

In patients with IBS, the mechanisms of action of rifaximin, beyond direct bactericidal effects, include decrement of host pro-inflammatory responses to bacterial products in patients with IBS, and has antibiotic efficacy against isolates derived from patients with small intestinal bacterial overgrowth [19,38,39,40].

On the other hand, patients with CP/CPPS traditionally receive empirical treatment, with the most common in clinical practice including antimicrobial agents and alpha-adrenergic receptor antagonists [32,38,41], or combination, multimodal therapy [39,42].

Our results fulfilled both primary outcomes, since we demonstrated that, in patients with diarrhoea-predominant IBS plus CP/CPPS, the administration of rifaximin followed by VSL#3 for a period of three months registered a reduction of ≥six points of the total NIH-CPSI score in 71.1% of patients with NIH IIIa prostatitis plus D-IBS. Notably, this value is higher than the placebo effect of ~64%, demonstrated in long-term studies [7]. At the same time, patients with IIIa prostatitis achieved as a result of therapy a significant improvement from baseline for IBS-SSS; this was associated with an IBS-SSS responder rate (in terms of decline >50 points) of 77.7%, significantly higher than the rate values found in IIIb patients (32.5%).

Results of the secondary endpoints were also noticeable, showing a better individual response at V3 in IIIa prostatitis (compared with IIIb prostatitis plus D-IBS or D-IBS alone) of urinary and gastrointestinal symptoms, and significant reduction of mean leukocyte counts on EPS after prostate massage in IIIa cases only.

The explanation of a better response on combined therapy in our study might be the complexity of IBS symptoms and natural disease course, requiring medications that might be effective against specific symptoms. These results suggest that patients with CP/CPPS plus D-IBS may have similar underlying pathophysiology, but they differ in severity.

The mechanisms that are not fully understood involve a genetic predisposition in the presence of a complex interaction of colonic cells, imbalance between commensal and pathogen bacteria of the gut microbiome, and local low-grade inflammation associated with IBS with abnormal immune function, gastrointestinal motility, and brain-gut interactions [40,41]. The resultant products of altered gut fermentation, termed ‘the fermentome’, can exist in the gaseous phase and are recognisable by volatile organic compounds (VOC) analysis in urine, breath, and faeces [41,43].

In patients with D-IBS burdened by the presence of comorbidities such as inflammatory prostatitis, we hypothesise that more severe urinary and intestinal symptoms in these patients may be secondary to an increased presence of VOC facilitated in part by increased intestinal permeability altered in certain gut diseases [43]. In this regard, ultrasound evaluation has recently revealed a significantly higher frequency of dilatation of the prostatic venous plexus (anatomical space between the posterior wall of the prostate and the anterior wall of the rectum) in patients with CBP plus IBS (75%) compared with patients with CBP alone (10%) [44].

Therefore, in our clinical model of patients, our adopted therapy (rifaximin followed by probiotic VSL#3) may have improved the urinary and gastrointestinal symptoms pattern through a likely reduction of the fermentome and VOC, at least in urine and faeces.

Although 49.4% of the patients (42 out of 85) showed clinical improvement in our study, there was a significant difference between the IIIa and IIIb groups, since a six-point or more reduction in total NIH-CPSI score was found respectively in 71% and 25%.

This huge difference may reflect the heterogeneous aspect of CP/CPPS mainly within subjects of category IIIb, which in a contemporary concept has a clinical presentation that is not prostate-specific but incorporates a phenotyping system with a varying number of positive domains among the following six: urinary, psychosocial, organ-specific, infection, neurologic/systemic, and tenderness (UPOINT), and a European study modified the clinical phenotyping system with an additional sexual dysfunction domain (UPOINTS) [45,46].

In May 2016, the new diagnostic criteria for functional bowel and anorectal disorders were defined in Rome IV criteria. The changes in criteria and new research findings might influence not only pathophisiological factors but also future treatments. Further studies are required to investigate these disorders [47,48].

## 5. Conclusions

The mechanisms of both commensal and pathogenic bacteria interaction with colonic cells and prostatitis are not fully understood. The treatment with rifaximin followed to probiotics reduces the urinary and gastrointestinal symptoms with a good compliance. Further studies with higher numbers of participants are necessary to confirm these results. 

## Figures and Tables

**Table 1:** Baseline characteristics of patients with chronic prostatitis (Type IIIa or IIIb) plus irritable bowel syndrome (IBS), or with IBS alone.

	Categories		
	Type IIIa Plus IBS	Type IIIb Plus IBS	IBS Alone
Patients (*n*)	45	40	75
Age (years)	30 (23–44)	29 (24–44)	30 (23–44)
BMI (kg/m^2^)	23 (19–28)	22 (20–28)	23 (21–28)
Time since diagnosis (months)	32 (20–60)	34 (22–58)	34 (24–50)
WBC on EPS after prostate massage	12 *^,^° (10–15)	7 ^†^ (4–10)	4 (2–6)

Irritable bowel syndrome = IBS; BMI = body mass index. WBC = white blood cells; EPS = expressed prostate secretion. Values were expressed as mean and range (in parentheses); * *p* < 0.01 vs. matched values of patients with IBS alone; ° *p* < 0.05 vs. matched values of patients with prostatitis type IIIb; ^†^ *p* < 0.05 vs. patients with IBS alone.

**Table 2:** Intragroup and intergroup analysis of National Institute of Health Chronic Prostatitis Symptom Index NIH-CPSI score and gastrointestinal symptoms in patients with chronic prostatitis (Type IIIa or IIIb plus IBS, or with IBS alone assessed before the treatment (V0) and three months afterward (V3).

	Categories					
	Type IIIa Plus IBS		Type IIIb Plus IBS		IBS Alone	
Study timepoint	V0 (*n* = 45)	V3 (*n* = 45)	V0 (*n* = 40)	V3 (*n* = 40)	V0 (*n* = 75)	V3 (*n* = 75)
Outcomes related to CP-CPPS						
Primary outcome						
NIH-CPSI responder rate (≥6 point decline) No./total No. (%)	NA	32/45 (71.1)	NA	10/40 (25)	NA	NA
Secondary outcomes						
WBC on EPS after prostate massage	12 *^,^° (10–15)	7 * (5–9)	7 ^†^ (4–10)	6 (4–9)	4 (2–6)	4 (2–6)
Prostatitis symptoms (NIH-CPSI score)						
Total score	21.2 (15–24)	14.5 * (9–19)	17.4 ° (10–21)	15.1 (13–18)	12.0 (6–14)	7.5 (3–10)
Pain subscale	11.9 (8–15)	8.5 * (5–11)	9.8 (8–11)	8.7 (7–11)	5.5 (4–7)	3.0 (2–5)
Urinary subscale	4.5 (3–6)	2.5 * (0–3)	3.6 (2–5)	3 (2–5)	3.5 (1–5)	2.0 (1–3)
Quality of life subscale	4.8 (3–7)	3.2 * (2–5)	4.0 (3–6)	3.4 (2–6)	4.0 (3–6)	2.5 (1–4)
Outcomes related to D-IBS						
Primary outcomes						
Mean IBS severity score	298.4 (180–410)	192.5 * (117–246)	270.0 (163–388)	198.5 (135–265)	262.5 (156–397)	204.7 (129–266)
IBSS responder rate (>50-point decline) No./total No. (%)	NA	35/45 (77.7)	NA	13/40 (32.5)	NA	NA
Secondary outcomes						
Gastrointestinal symptoms						
Abdominal pain	48.6 (25–63)	25.5 * (10–35)	40.5 ° (21–65)	28.5 * (12–40)	38.5 ° (18–58)	30.5 * (11–43)
Frequency of abdominal pain	50 (28–75)	35.0 * (30–55)	48.5 ° (30–70)	38.5 * (22–60)	45.0 ° (35–70)	35.0 * (30–67)
Abdominal distension/bloating	44.5 (35–74)	25.5 * (18–35)	39 (30–67)	28.5 * (15–40)	42 (30–85)	28.5 * (15–38)
Dissatisfaction with bowel habits	78.0 (62–94)	50.5 * (40–74)	75.0 (66–100)	55.0 (40–82)	75.0 (60–95)	55.0 (35–78)
Interference with quality of life	77.0 (60–105)	38.0 * (22–47)	68.0 (15–87)	48.0 (15–25)	66.0 (15–89)	55.7 (33–77)

Irritable bowel syndrome = IBS; National Institute of Health Chronic Prostatitis Symptom Index (NIH-CPSI); No. = number; IBS Severity Scoring System = IBS-SSS; Values were expressed as mean and range (in parentheses); NA = not applicable; * *p* < 0.05 vs. pre-treatment matched values; ° *p* < 0.05 vs. matched values of patients with prostatitis type IIIa; ^†^ *p* < 0.05 vs. patients with IBS alone.
